# Editorial: Multiscale computational approaches in infectious diseases

**DOI:** 10.3389/fmicb.2022.947673

**Published:** 2022-07-29

**Authors:** Esteban A. Hernandez-Vargas, Jorge X. Velasco-Hernández, Dunja Bruder

**Affiliations:** ^1^Instituto de Matemáticas, Universidad Nacional Autónoma de México, Querétaro, Mexico; ^2^Institute of Medical Microbiology and Hospital Hygiene, Otto-von-Guericke University Magdeburg, Magdeburg, Germany

**Keywords:** mathematical modeling, multiscale approach, infectious diseases, computational modeling, biological process

Infectious diseases are a dynamic composition of interrelated activities at different spatial and temporal scales (Hernandez-Vargas et al., 2019). Their complexity is one of their most pervasive and dominant characteristics. To understand these complex mathematical methods, exist that constitute a methodological tool that incorporates a large variety of techniques and data encompassing many of the classic and new areas of mathematical inquiry. The present Research Topic explores the application of so-called multiscale mathematical and computational to study the dynamics of some biological systems involved in infectious diseases. The analysis of biological processes requires multidisciplinary work since, as mentioned above, molecular, cellular, and ecological mechanisms may be present and play an important role in defining the observable behavior and function of a given process. Thus, multiscale mathematical models aim to postulate mechanisms that structure the diversity of scales interacting based on specific results coming from laboratory, field, or even clinical studies.

A “Frontiers in Immunology” and “Frontiers in Microbiology” Research Topic was proposed to address the current state of the art of multiscale computational approaches covering processes at multiple temporal and/or spatial scales (e.g., genes, molecular, cells, tissues, organs, individual, and population) and in combination with animal experiments and clinical data.

A total of seven papers were accepted for publication, which attests to the timeliness of the Research Topic. At the within-host level, the first paper (Hooker and Ganusov) considers a computational analysis showing that the antiviral oseltamivir can impact influenza kinetics at the end of viral shedding, and in about 20–40% of volunteers that shed the virus, treatment had no impact on viral shedding duration.

The second paper (Millar et al.) considers a systems biology approach at a within-host level to dissect the structural organization of granulomas, as well as the recruitment of non-specific T cells likely, which may contribute to reduced responsiveness to Tuberculosis.

The third article (Nurjadi et al.) revealed that careful analysis of the whole genome data and additional criteria such as lineage-independent mutations in *Staphylococcus aureus* may be useful for the identification of mutations leading to phenotypic resistance.

The fourth article (Dimas Martins and Gjini) proposes a mathematical framework based on the Lotka-Volterra model to capture the frequency-dependent competition between microbial strains within-host and upon transmission. As a proof-of-concept, the model is applied to a dataset from *in-vivo* competitive mixture experiments with influenza strains in ferrets.

The fifth article (Blickensdorf et al.) presents a hybrid agent-based infection model to quantitatively compare different scenarios and discuss the importance of Pores of Kohn during infections of *Aspergillus fumigatus* (an airborne opportunistic fungus). Simulations revealed that the Pores of Kohn alter important infection clearance mechanisms, such as the spatial distribution of macrophages and the effect of chemokine signaling.

The sixth article (Arias-del-Angel et al.) shows infection-kinetics experiments on various cell lines and developed a mathematical model to simulate the experimental outcomes. Results suggest that a process related to the cell replication rate may strongly influence the parasite invasion efficiency.

The seventh article (Mascheroni et al.) presents tumor-targeting bacteria that elicit anticancer effects by infiltrating hypoxic regions, releasing toxic agents, and inducing immune responses. Simulations show that active bacterial migration toward tumor hypoxic regions provides optimal infiltration and that high killing rates combined with high chemotactic values provide the smallest tumor volumes at the end of the treatment.

These papers provide a broad overview of current issues to model different infectious diseases. We would like to thank the Frontiers Editorial Staff, all the authors who contributed excellent papers, as well as the reviewers whose work has made the publication of this Research Topic possible.

## Author contributions

All authors listed have made a substantial, direct, and intellectual contribution to the work and approved it for publication.

## Conflict of interest

The authors declare that the research was conducted in the absence of any commercial or financial relationships that could be construed as a potential conflict of interest.

## Publisher's note

All claims expressed in this article are solely those of the authors and do not necessarily represent those of their affiliated organizations, or those of the publisher, the editors and the reviewers. Any product that may be evaluated in this article, or claim that may be made by its manufacturer, is not guaranteed or endorsed by the publisher.
